# On the Ribosomal Density that Maximizes Protein Translation Rate

**DOI:** 10.1371/journal.pone.0166481

**Published:** 2016-11-18

**Authors:** Yoram Zarai, Michael Margaliot, Tamir Tuller

**Affiliations:** 1 School of Electrical Engineering, Tel-Aviv University, Tel-Aviv 69978, Israel; 2 School of Electrical Engineering and the Sagol School of Neuroscience, Tel-Aviv University, Tel-Aviv 69978, Israel; 3 Dept. of Biomedical Engineering and the Sagol School of Neuroscience, Tel-Aviv University, Tel-Aviv 69978, Israel; John Curtin School of Medical Research, AUSTRALIA

## Abstract

During mRNA translation, several ribosomes attach to the same mRNA molecule simultaneously translating it into a protein. This pipelining increases the protein translation rate. A natural and important question is what ribosomal density maximizes the protein translation rate. Using mathematical models of ribosome flow along both a linear and a circular mRNA molecules we prove that typically the steady-state protein translation rate is maximized when the ribosomal density is one half of the maximal possible density. We discuss the implications of our results to endogenous genes under natural cellular conditions and also to synthetic biology.

## Introduction

The transformation of the genetic information in the DNA into functional proteins is called *gene expression*. Two important steps in gene expression are *transcription* of the DNA code into messenger RNA (mRNA) by RNA polymerase (RNAP), and then *translation* of the mRNA into proteins. During translation, complex macromolecules called ribosomes traverse the mRNA strand, decoding it codon by codon into a corresponding chain of amino-acids that is folded co- and post-translationally to become a functional protein [1]. The rate in which proteins are produced during the translation step is called the protein translation rate (or protein production rate).

According to current knowledge, translation takes place in all living organisms and under all conditions. Understanding the numerous factors that affect this dynamical process has important implications to many scientific disciplines including medicine, evolutionary biology, synthetic biology, and more.

Computational models of translation are becoming increasingly important as the amount of experimental findings related to translation rapidly increases (see, e.g. [2–11]). Such models are particularly important in the context of synthetic biology and biotechnology, as they can provide predictions on the qualitative and quantitative effects of various manipulations of the genetic machinery. Recent advances in measuring translation in *real time* [12–15] will probably further increase the interest in computational models that can integrate and explain the measured biological data.

During translation, a large number of ribosomes act simultaneously on the same mRNA molecule. This pipelining of the protein translation leads to a more continuous translation rate and increased efficiency. Indeed, the translation rate may reach five new peptide bonds per second in eukaryotes, and 15 new bonds in prokaryotes [16].

The ribosomal density along the mRNA molecule may affect different fundamental intracellular phenomena. A very high density can lead to ribosomal traffic jams, collisions and abortions (see, e.g. [17]). It may also contribute to co-translational misfolding of proteins. On the other hand, a very low ribosomal density may lead to a low translation rate, and a high degradation rate of mRNA molecules [18–24]. Thus, a natural and important question is what ribosomal density optimizes one (or more) biological properties, for example, the protein translation rate. Optimizing the protein translation rate is also an important challenge in synthetic biology and biotechnology, where a standard objective is to maximize the translation efficiency and protein levels of heterologous genes in a new host (see, e.g., chapter 9 in [25]).

In this paper, we analyze the density that maximizes the protein translation rate using a mathematical model of ribosome flow along the mRNA molecule. A standard mathematical model for ribosome flow is the *totally asymmetric simple exclusion process* (TASEP) [26, 27]. In this model, particles hop unidirectionally along an ordered lattice of *L* sites. Every site can be either free or occupied by a particle, and a particle can only hop to a free site. This simple exclusion principle models particles that have “volume” and thus cannot overtake one other. The hops are stochastic, and the rate of hoping from site *i* to site *i* + 1 is denoted by *γ*_*i*_. A particle can hop to [from] the first [last] site of the lattice at a rate *α* [*β*]. The average flow through the lattice converges to a steady-state value that depends on the parameters *L*, *α*, *γ*_1_, …, *γ*_*L*−1_, *β*. Analysis of TASEP is non trivial, and closed-form results have been obtained mainly for the homogeneous TASEP (HTASEP), i.e. for the case where all the *γ*_*i*_s are assumed to be equal.

TASEP has become a fundamental model in non-equilibrium statistical mechanics, and has been applied to model numerous natural and artificial processes [28]. In the context of translation, the lattice models the mRNA molecule, the particles are ribosomes, and simple exclusion means that a ribosome cannot overtake a ribosome in front of it.

TASEP has two standard configurations. In TASEP with *open boundary conditions* the two sides of the chain are connected to two particle reservoirs, and particles can hop into the chain (if the first site is empty) and out of the chain (if the last site is full). In TASEP with *periodic boundary conditions* the chain is closed, and a particle that hops from the last site returns to the first one. Thus, here the particles hop around a ring, and the number of particles on the ring is conserved.

**Fig 1:**
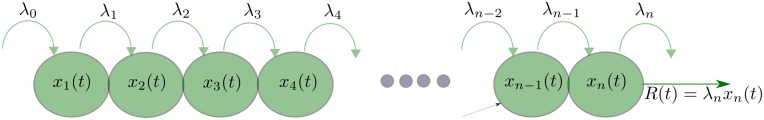
The RFM models unidirectional flow along a chain of *n* sites. The state variable *x*_*i*_(*t*) ∈ [0,1] describes the density of site *i* at time *t*. The parameter *λ*_*i*_ > 0 controls the transition rate from site *i* to site *i* + 1, with *λ*_0_ [*λ*_*n*_] controlling the initiation [exit] rate. The ribosome output rate at time *t* is *R*(*t*) = *λ*_*n*_ *x*_*n*_(*t*). This is the amount of proteins produced per time unit on the modeled mRNA molecule.

The *ribosome flow model* (RFM) [29] is a continuous-time, deterministic, compartmental model for the unidirectional flow of “material” along an open chain of *n* consecutive compartments (or sites). The RFM can be derived via a dynamic mean-field approximation of TASEP with open boundary conditions (see section 4.9.7 in [28] and page R345 in [30]). The RFM includes *n* state variables, denoted *x*_1_(*t*), …*x*_*n*_(*t*), with *x*_*i*_(*t*) describing the amount (or density) of “material” in site *i* at time *t*, normalized such that *x*_*i*_(*t*) = 1 [*x*_*i*_(*t*) = 0] indicates that site *i* is completely full [completely empty] at time *t*. In the RFM, the two sides of the chain are connected to two particle reservoirs. A parameter *λ*_*i*_ > 0, *i* = 0, …, *n*, controls the transition rate from site *i* to site *i* + 1, where *λ*_0_ [*λ*_*n*_] is the initiation [exit] rate (see Fig 1).

In the *ribosome flow model on a ring* (RFMR) [31] the particles exiting the last site enter the first site. This is the dynamic mean-field approximation of TASEP with periodic boundary conditions. The RFMR admits a first integral, i.e. a quantity that is preserved along the dynamics, as the total density is conserved. Both the RFM and RFMR are cooperative dynamical systems [32], but their dynamical properties turn out to be quite different [31].

The RFM [RFMR] has been applied to model and analyze ribosome flow along an open [circular] mRNA molecule during translation. Indeed, in eukaryotes the mRNA is often temporarily circularized (via non-covalent interactions), for example, by translation initiation factors [33]. Thus, a large fraction of the ribosomes that complete translating the mRNA re-initiate, and thus the RFMR is a good approximation of the translation dynamics in these circularized mRNAs. In addition, circular RNA forms (which include covalent RNA interactions) appear in all domains of life [34–41]. Specifically, it was recently suggested that circular RNAs can be translated in eukaryotes [39–41]. These cases are better approximated by the RFMR. Note that there are also cases of circular DNA [42, 43]. This issue is not directly related to translation, yet transcription of such circular DNAs may be analyzed by a model similar to the RFMR [44] (see also [45]).

**Fig 2:**
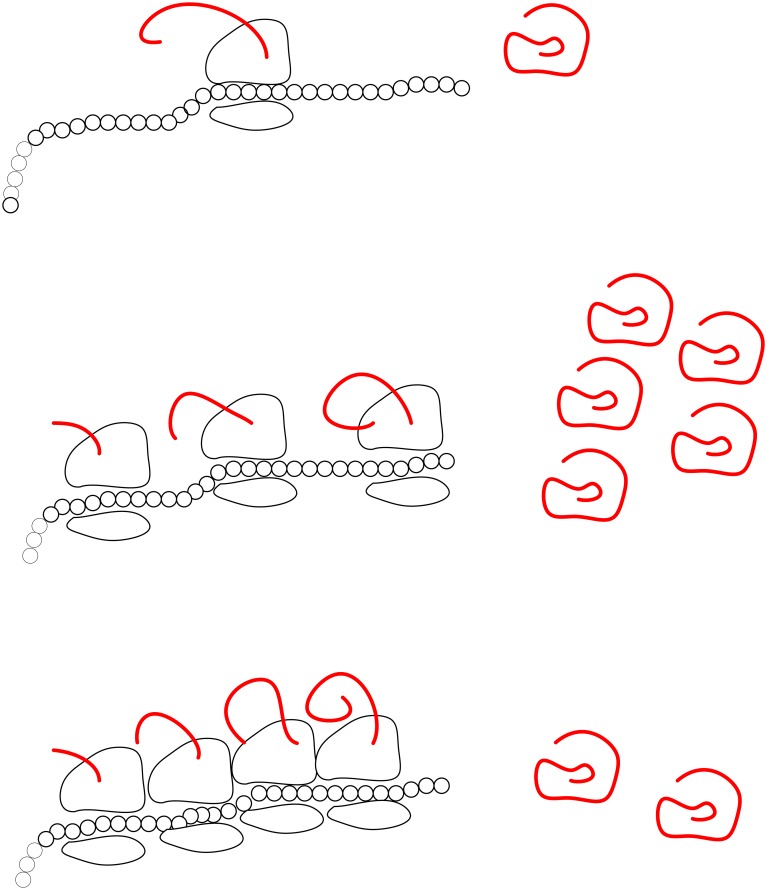
Protein translation rate and ribosome density: too few ribosomes (upper figure) lead to a low translation rate, as do too many ribosomes (lower figure) due to traffic jams along the mRNA. Optimal translation is achieved when the total density is one half of the maximal possible total density (middle figure).

Here, we use the RFM [RFMR] to analyze the ribosomal density along a linear [circular] mRNA molecule that maximizes the steady-state protein translation rate. We refer to this density as the *optimal density*. This problem has already been studied before. For example, Zouridis and Hatzimanikatis [46] derived a deterministic, sequence-specific kinetic model for translation and studied the effect of the average ribosomal density on the steady-state translation rate. Their model assumes homogeneous elongation rates and open-boundary conditions, and includes all the elementary steps involved in the elongation cycle at every codon. Their simulations suggest that there exists a unique average density that corresponds to a maximal translation rate, see Figures 2A and 5A in [46] (see also [47]).

The RFM and RFMR are simpler models and thus allow to rigorously prove several analytic results on the optimal density. For a circular mRNA, we prove that there always exists a unique optimal density that maximizes the steady-state translation rate, and that it can be determined efficiently using a simple “hill climbing” algorithm. In addition, we show that under certain symmetry conditions on the rates the optimal density is one half of the maximal possible density.

In the case of a linear mRNA molecule, we prove that when the initiation and elongations rates are chosen to maximize the translation rate, under an affine constraint on the rates, the corresponding optimal density is one half of the maximal possible density (see Fig 2).

The remainder of this paper is organized as follows. The next section briefly reviews the RFM and the RFMR. Section 2 describes our main results. Section 3 summarizes the results, describes their biological implications, and suggests several directions for further research. The mathematical background and details are given in Appendix A. The proofs of all the results are placed in Appendix B.

## 1 The Ribosome Flow Model

Recall that the RFM models the ribosomes on the mRNA as “material” that flows between consecutive sites. For each site, the dynamics of the model can be expressed mathematically by the change in the amount of the material (i.e. ribosomes density) in that site, as a function of time, which is simply equal to the input rate to that site minus the output rate from that site. Since the model contains *n* sites, the RFM is expressed by *n* first-order ordinary differential equations describing the change in the amount of material in each site:

$$\begin{array}{lll} {\dot{x}}_{1} & = & \lambda_{0}\left(1-x_{1}\right)-\lambda_{1}x_{1}\left(1-x_{2}\right),\\ {\dot{x}}_{2} & = & \lambda_{1}x_{1}\left(1-x_{2}\right)-\lambda_{2}x_{2}\left(1-x_{3}\right),\\ {\dot{x}}_{3} & = & \lambda_{2}x_{2}\left(1-x_{3}\right)-\lambda_{3}x_{3}\left(1-x_{4}\right),\\ & \vdots & \\ {\dot{x}}_{n-1} & = & \lambda_{n-2}x_{n-2}\left(1-x_{n-1}\right)-\lambda_{n-1}x_{n-1}\left(1-x_{n}\right),\\ {\dot{x}}_{n} & = & \lambda_{n-1}x_{n-1}\left(1-x_{n}\right)-\lambda_{n}x_{n}, \end{array}$$ (1)

where ${\dot{x}}_{i}$ denote the change in the amount of material in site *i* as a function of time, i.e. ${\dot{x}}_{i}≔\frac{d}{dt}x_{i}\left(t\right)$, *i* = 1, …, *n*. Note that this is a set of *n* nonlinear ordinary differential equations. If we define *x*_0_(*t*) ≔ 1 and *x*_*n*+1_(*t*) ≔ 0 then Eq (1) can be written more succinctly as

$${\dot{x}}_{i}=\lambda_{i-1}x_{i-1}\left(1-x_{i}\right)-\lambda_{i}x_{i}\left(1-x_{i+1}\right),\;i=1,\cdots ,n.$$ (2)

This can be explained as follows. The input rate to site *i* is *λ*_*i* − 1_ *x*_*i* − 1_(*t*)(1 − *x*_*i*_(*t*)). This flow is proportional to *x*_*i* − 1_(*t*), i.e. it increases with the density at site *i* − 1, and to (1 − *x*_*i*_(*t*)), i.e. it decreases as site *i* becomes fuller. In particular, when this site is completely full, i.e. *x*_*i*_(*t*) = 1, there is no flow into this site. This corresponds to a “soft” version of a simple exclusion principle: the flow of particles into a site decreases as that site becomes fuller. Note that the maximal possible flow from site *i* − 1 to site *i* is the (*i* − 1)th transition rate *λ*_*i* − 1_. Similarly, the output rate from site *i*, which is also the input rate to site *i* + 1, is given by *λ*_*i*_ *x*_*i*_(*t*)(1 − *x*_*i* + 1_(*t*)).

The output rate of ribosomes from the chain is *R*(*t*) ≔ *λ*_*n*_ *x*_*n*_(*t*), that is, the flow out of the last site. This is the number of proteins generated per time unit while translating the modeled mRNA template.

Suppose that we fix the values of the parameters in the RFM, that is, the transition rates. For any set of initial densities along the mRNA at the initial time zero, that is, the vector *x*(0) = [*x*_1_(0) … *x*_*n*_(0)]^*T*^, the RFM admits a unique dynamical solution *x*(*t*) = [*x*_1_(*t*) … *x*_*n*_(*t*)]^*T*^ for any time *t*, where *x*_*i*_(*t*) is the density at site *i* at time *t*. Since every *x*_*i*_ describes amount of normalized material in the sites, we always assume that their initial values are between zero and one. Combining this with the RFM dynamics, it can be shown that the amount of material in each site is bounded between zero and one *for all time*, i.e. *x*_*i*_(*t*) ∈ [0,1], for all *t* ≥ 0 (see Section A.1). We denote the state-space, i.e. the space of all possible values of the state variables, by *C*^*n*^.

It was shown in [48] that the RFM is a *cooperative dynamical system* [32], and that this implies the following property. Every solution of the dynamics converges to a steady-state value, that is, the density *x*_*i*_(*t*) at site *i* converges, as time goes to infinity, to a specific value *e*_*i*_. We denote *e* ≔ [*e*_1_ … *e*_*n*_]^*T*^, i.e. the column vector of steady-state densities at each site. Furthermore, the value *e* will not depend on the initial value *x*(0). This means that if we simulate the RFM starting from any density of ribosomes on the mRNA the dynamics will always converge to the same final state (i.e., the same final ribosome density along the mRNA). Mathematically, the convergence property is written as lim_*t*→∞_ *x*(*t*, *x*(0)) = *e* for all *x*(0) ∈ *C*^*n*^ (see also [49]). In particular, since *x*_*n*_(*t*) converges to *e*_*n*_, the translation rate *R*(*t*) = *λ*_*n*_ *x*_*n*_(*t*) converges to the steady-state value:

$$R≔\lambda_{n}e_{n}.$$ (3)

This steady-state solution, that depends on all the rates but not on the initial state of the chain, is analyzed below. The value of *e* does depend on the rates, i.e. *e* = *e*(*λ*_0_, …, *λ*_*n*_) and satisfies *e*_*i*_ ∈ (0,1) for all *i*, that is, at steady-state each site is not completely empty nor completely full.

### 1.1 Ribosome Flow Model on a Ring

If we consider the RFM under the additional assumption that all the ribosomes leaving site *n* circulate back to site 1 then we obtain the RFMR. Just like the RFM, this is described by *n* nonlinear, first-order ordinary differential equations:

$$\begin{array}{lll} {\dot{x}}_{1} & = & \lambda_{n}x_{n}\left(1-x_{1}\right)-\lambda_{1}x_{1}\left(1-x_{2}\right),\\ {\dot{x}}_{2} & = & \lambda_{1}x_{1}\left(1-x_{2}\right)-\lambda_{2}x_{2}\left(1-x_{3}\right),\\ & \vdots & \\ {\dot{x}}_{n} & = & \lambda_{n-1}x_{n-1}\left(1-x_{n}\right)-\lambda_{n}x_{n}\left(1-x_{1}\right). \end{array}$$ (4)

Note that the only difference here relative to the RFM is in the equations describing the change of material in sites 1 and *n*. Specifically, the flow out of site *n* is the flow into site 1. This model assumes perfect re-cycling, but it also provides a good approximation when a large fraction of the ribosomes are re-cycled. Note that the model here is indifferent to the nature of the biochemical nature of the mRNA circularization; for example it can be both covalent or non-covalent RNA.

The RFMR can also be written succinctly as Eq (2), but now with every index interpreted modulo *n*. In particular, *λ*_0_ [*x*_0_] is replaced by *λ*_*n*_ [*x*_*n*_].

The total density of ribosomes along the chain at time *t* is given by

$$H\left(x\left(t\right)\right)≔x_{1}\left(t\right)+\cdots +x_{n}\left(t\right),$$

i.e. the sum of the density at each site. Also, let *s* denote the total density of ribosomes along the chain at time 0, *s* ≔ *H*(*x*(0)). In the RFM, ribosomes enter and leave the chain and therefore *H*(*x*(*t*)) may vary with time *t*. In the RFMR, ribosomes that exit site *n* circulate back to site 1, so the total density is preserved for all time. This means that *H*(*x*(*t*)) = *s* for all *t* ≥ 0. The dynamics of the RFMR thus redistributes the particles between the sites, but without changing the total ribosome density. In the context of translation, this means that the total number of ribosomes on the (circular) mRNA is conserved. Mathematically, this means that *H*(*x*(*t*)) is a *first integral* of the RFMR (see Section A.2).

To understand the dynamics of the RFMR, we denote by *L*_*s*_ the set of all possible ribosome density configurations such that the total density is equal to *s*. For example, *L*_*s*_ includes the configuration where site 1 has a density *s* and all other sites are empty. It also includes the configuration where sites 1 and 2 have a density *s*/2 and all other sites are empty, etc. Mathematically, for *s* ∈ [0,*n*], the *s level set* of *H* is

$$L_{s}≔\left\{y\in C^{n}:1_{n}^{T}y=s\right\},$$

where for p∈R, *p*_*n*_ denotes the column vector ${\left[\begin{array}{cccc} p & p & \ldots & p \end{array}\right]}^{T}\in {\mathbb{R}}^{n}$.

Ref. [31] has shown that the RFMR is a strongly cooperative dynamical system, that every level set *L*_*s*_ contains a unique equilibrium point *e* = *e*(*s*, *λ*_1_, …, *λ*_*n*_), and that any trajectory of the RFMR emanating from any *x*(0) ∈ *L*_*s*_ converges to this equilibrium point. In particular, the translation rate converges to the steady-state value *R* = *R*(*s*, *λ*_1_, …, *λ*_*n*_) (see Section A.2). This means the following. Fix an arbitrary value *s* ∈ [0,*n*]. Then the RFMR, initiated with any configuration with total density *s*, will converge to the same final steady-state density *e*. This final density depends on the rates and the value *s*. For example if *s* = 0, corresponding to the initial condition *x*(0) = 0_*n*_, then *x*(*t*) ≡ 0_*n*_ for all *t* ≥ 0, so *e* = 0_*n*_. Similarly, *s* = *n* corresponds to the initial condition *x*(0) = 1_*n*_ and then clearly *x*(*t*) ≡ 1_*n*_ for all *t* ≥ 0, so *e* = 1_*n*_. Since these two cases are trivial, below we will always assume that *s* ∈ (0, *n*). In this case, *e* ∈ Int(*C*^*n*^), that is, the final density in each site will be strictly larger than zero and strictly smaller than one.

For more on the analysis of the RFM and the RFMR using tools from systems and control theory, see [31, 50–54]. For a general discussion on using systems and control theory in systems biology, see the excellent survey papers by Sontag [55, 56].

The RFM models translation on a single isolated mRNA molecule. A network of RFMs, interconnected through a common pool of “free” ribosomes has been used to model simultaneous translation of several mRNA molecules while competing for the available ribosomes [57]. It is important to note that many analysis results for the RFM, RFMR, and networks of RFMs hold for any set of transition rates. This is in contrast to the analysis results on the TASEP model. Rigorous analysis of TASEP seems to be tractable only under the assumption that the internal hopping rates are all equal (i.e. the homogeneous case). In the context of translation, this models the case where all elongation rates are assumed to be equal.

The next section describes our main results on the optimal ribosome density.

## 2 Main Results

The average ribosomal density along the mRNA molecule at time *t* is simply the sum of all the site densities at time *t*, divided by the number of sites. We denote this average density by $\rho \left(t\right)≔\frac{1}{n}\left(x_{1}\left(t\right)+\ldots +x_{n}\left(t\right)\right)$. Recall that for every set of parameters in our models the state variables converge to a steady-state *e*. In particular, *ρ*(*t*) converges to the steady-state average ribosomal density:

$$\rho ≔\frac{1}{n}\left(e_{1}+\cdots +e_{n}\right).$$

Note that since *e*_*i*_ ∈ [0,1] for all *i*, *ρ* ∈ [0,1]. We are interested in analyzing the density that is obtained when the parameter values in the model are the ones that maximize the steady-state translation rate. Our results below show that there is a correspondence between the optimal translation rate and the optimal average ribosomal density: the optimal translation rate is obtained when and only when the average ribosomal density admits a specific value.

### 2.1 Optimal Density in the RFMR

Recall that in the RFMR the dynamical behavior depends on the transition rates and on the total density of ribosomes on the mRNA at time zero $s≔\sum_{i=1}^{n}x_{i}\left(0\right)$ that, due to conservation of the total number of ribosomes, is equal to the total density at any time *t*. This means that the average ribosomal density is constant: *ρ*(*t*) ≡ *s*/*n*.

Our first result shows that in the case of a circular mRNA there always exists a *unique* average density of ribosomes ρ* = s*/*n* that corresponds to a maximal steady-state translation rate of proteins. This means that in order to maximize the steady-state translation rate, the mRNA must be initialized with a total density s* (the distribution of this total density along the mRNA at time zero is not important). Initializing with either more or less than s* will decrease the steady-state translation rate with respect to the one obtained when the circular mRNA is initialized with total density s*. The optimal value s* depends on the transition rates. This is mathematically formulated as follows. Fix arbitrary transition rates *λ*_*i*_ > 0, *i* = 1, …, *n*, and let *R*(*s*) ≔ *R*(*s*;*λ*_1_, …, *λ*_*n*_) and *e*(*s*) ≔ *e*(*s*;*λ*_1_, …, *λ*_*n*_) denote the steady-state translation rate and the steady-state ribosomal density profile, respectively, as a function of *s*.

**Proposition 1** *For any set of rates λ*_*i*_ > 0 in the RFMR there exists a unique value s* = s*(*λ*_1_, …, *λ*_*n*_) ∈ (0, *n*) *that maximizes R*(*s*). Furthermore, for this optimal value e* ≔ *e*(s*) and R* ≔ *R*(s*) *satisfy*

$$e_{1}^{*}\cdots e_{n}^{*}=\left(1-e_{1}^{*}\right)\cdots \left(1-e_{n}^{*}\right),$$ (5)

*and*

$${\left(R^{*}\right)}^{n}=\left(\lambda_{1}\cdots \lambda_{n}\right){\left(e_{1}^{*}\cdots e_{n}^{*}\right)}^{2}.$$ (6)

The optimality condition (5) can be explained as follows. If the total ribosome density *s* is very small then there will not be enough ribosomes on the circular mRNA and the translation rate will be small (for example, for *s* = 0 we have *e* = 0_*n*_, and thus *R* = *λ*_1_*e*_1_(1 − *e*_2_) = 0). In this case, the product of the *e*_*i*_s is small, so *e*_1_…*e*_*n*_ < (1 − *e*_1_)…(1 − *e*_*n*_) and Eq (5) does not hold. If *s* is very large traffic jams evolve on the mRNA and again the translation rate will be small (for example, for *s* = *n* we have *e* = 1_*n*_, and thus *R* = *λ*_1_*e*_1_(1 − *e*_2_) = 0). In this case, *e*_1_…*e*_*n*_ > (1 − *e*_1_)…(1 − *e*_*n*_) and Eq (5) does not hold. Thus, Eq (5) describes the unique point where the balance between too few and too many ribosomes on the circular mRNA molecule is optimal.

The proof of this result (given in Appendix B) shows that the steady-state translation rate *R*(*s*) is strictly increasing on [0,s*) and strictly decreasing on (s*, *n*], so a simple “hill climbing” algorithm can be used to find s*.

The next example demonstrates Proposition 1 in the special case where all the elongation rates along the circular mRNA are identical. In this case, as shown in the example, the average ribosome density that maximizes the steady-state protein translation rate is exactly one half of the maximal possible total density.

**Example 1** Consider an RFMR with *λ*_1_ = … = *λ*_*n*_, i.e. all the elongation rates are equal. Denote their common value by *λ*_*c*_. Then it follows from Eq (4) that 1_*n*_ *c*, *c* > 0, is an equilibrium point. In other words, any density profile with an identical density *c* at each site is a steady-state. Consider an initial condition with total density *s*. Then the trajectory satisfies *x*_1_(*t*) + … + *x*_*n*_(*t*) = *s* for all *t* ≥ 0. In particular, this must hold for the steady-state value, so *nc* = *s* or *c* = *s*/*n*. By uniqueness of the equilibrium point in every level set of *H* this implies that *e* = (*s*/*n*)1_*n*_, so *R* = *λ*_*n*_*e*_*n*_(1 − *e*_1_) = *λ*_*c*_(*s*/*n*)(1 − (*s*/*n*)). Thus, $\frac{\partial R}{\partial s}=\frac{\lambda_{c}}{n^{2}}\left(n-2s\right)$, so *R*(*s*) is strictly increasing [decreasing] on *s* ∈ [0,*n*/2] [*s* ∈ [*n*/2, *n*]] and therefore attains a unique maximum at s* = *n*/2. Then e* ≔ *e*(s*) = (1/2)1_*n*_ and R* ≔ *R*(s*) = *λ*_*c*_/4, and it is straightforward to verify that Eqs (5) and (6) hold. Note also that $\frac{\partial^{2}R}{\partial s^{2}}=-\frac{2\lambda_{c}}{n^{2}}<0$, implying that *R*(*s*) is a strictly concave function.

The next example demonstrates the dependence of *R*(*s*) on *s* when the rates are not homogeneous.

**Fig 3:**
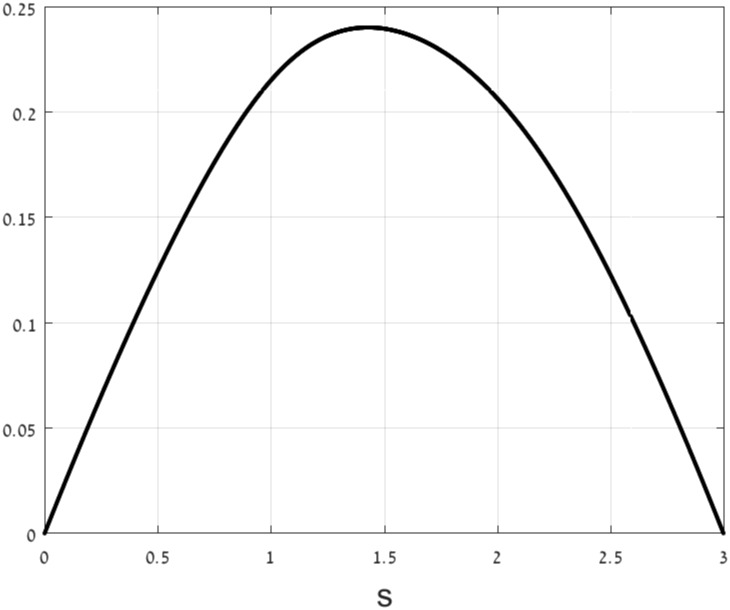
Steady-state protein translation rate *R*(*s*) as a function of *s* for the RFMR in Example 2. Here *s* is the total density of ribosomes along the circular mRNA.

**Fig 4:**
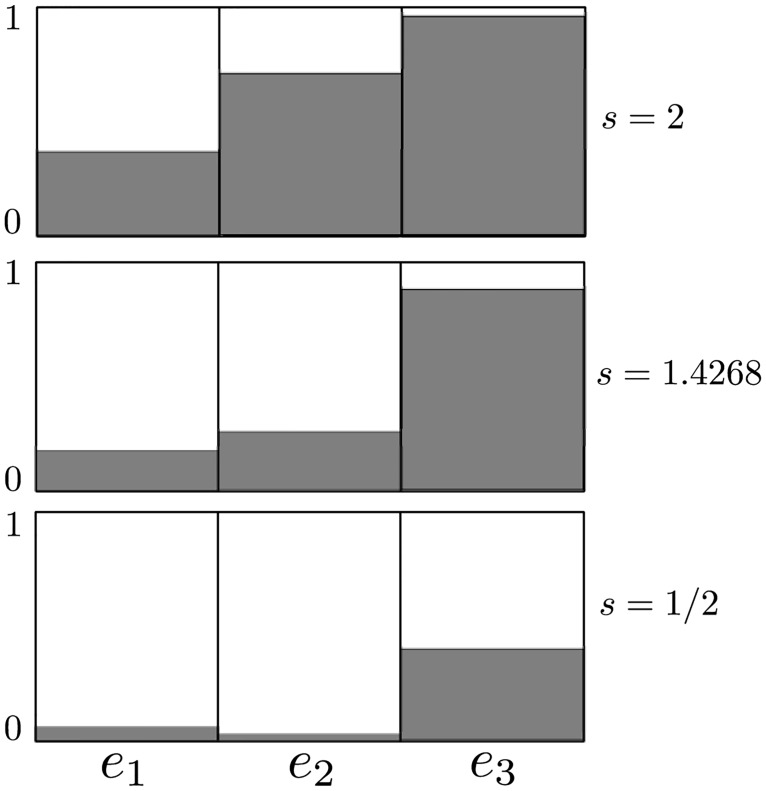
Equilibrium point *e* for the RFMR in Example 2 with *n* = 3 i.e. three sites, and transition rates *λ*_1_ = 2, *λ*_2_ = 6, *λ*_3_ = 1/3 for three different *s* values. Here *s* is the total density of ribosomes on the molecule, and *e* is the vector of steady-state densities in the three sites of the RFMR. The density in the third site (*e*_3_) is the highest in all cases, as the transition rate into [out of] this site is the highest [lowest].

**Example 2** Consider an RFMR with dimension *n* = 3 and transition rates *λ*_1_ = 2, *λ*_2_ = 6, and *λ*_3_ = 1/3. Fig 3 depicts *R*(*s*) for *s* ∈ [0,3]. It may be seen that *R*(*s*) attains a unique maximum at s* = 1.4268 (all numerical values in this paper are to four digit accuracy). The corresponding equilibrium point is e* = [0.1862 0.3539 0.8867]^*T*^, and the optimal translation rate is $R^{*}=\lambda_{1}e_{1}^{*}\left(1-e_{2}^{*}\right)=0.2405$. Fig 4 depicts a histogram of the equilibrium point *e* for three values of the level set parameter: *s* = 1/2, *s* = 1.4268, and *s* = 2. Note that *e*_3_ is the maximal entry in *e* for all *s*. This is due to fact that the entry rate *λ*_2_ = 6 into site 3 is high, and the exit rate *λ*_3_ = 1/3 from site 3 is low.

In other words, in the typical case where the elongation rates along the circular mRNA are not all identical the average ribosome density that maximizes the protein translation rate is *v*ery close but not equal to half of the maximal possible total density. See Section A.3 for more explanations on Fig 4.

For small values of *n* it is possible to provide more explicit results. The following result provides the maximal possible translation rate value, as a function of the elongation rates, in a very short RFMR with 2 or 3 sites.

**Fact 1** *For an RFMR with n* = 2 the optimal values are s* = 1 *and*

$$R^{*}=\frac{\lambda_{1}\lambda_{2}}{{\left(\sqrt{\lambda_{1}}+\sqrt{\lambda_{2}}\right)}^{2}}.$$ (7)

*For an RFMR with n* = 3 *the optimal translation rate satisfies the equation:*

$$2\sqrt{\lambda_{1}\lambda_{2}\lambda_{3}}{\left(R^{*}\right)}^{3/2}+\left(\lambda_{1}\lambda_{2}+\lambda_{1}\lambda_{3}+\lambda_{2}\lambda_{3}\right)R^{*}-\lambda_{1}\lambda_{2}\lambda_{3}=0.$$ (8)

It is interesting to understand how the steady-state densities along the mRNA change as the total density along the circular mRNA varies slightly from the optimal value s*. This is important since in practice achieving a total number of ribosomes that equals exactly s* may be difficult, and the actual total number may be close, but not exactly equal to s*. For fixed transition rates, let $e_{i}^{\prime }≔\frac{\partial }{\partial s}e_{i}\left(s\right)$ denote the change in the steady-state ribosome density at site *i* corresponding to a small change in the the total density *s*. We refer to $e_{i}^{\prime }$ as the sensitivity of *e*_*i*_ with respect to a change in the total density *s*.

The next result provides an expression for these sensitivities at the optimal density (i.e. the one that maximizes the protein translation rate). It can be used to compute how increasing/decreasing the total number of ribosomes on the circular mRNA affects the optimal density profile.

**Proposition 2** *Consider an RFMR with dimension n*. *Fix rates λ*_*i*_ > 0, and let s* = s*(*λ*_1_, …, *λ*_*n*_) *and* e* = e*(*λ*_1_, …, *λ*_*n*_) *be as defined in Proposition 1*. *Then* ${\left(e^{*}\right)}^{\prime }=\frac{v}{1_{n}^{T}v}$, *where*

$$v≔{\left[\begin{array}{ccccc} \frac{e_{1}^{*}\cdots e_{n-1}^{*}}{\left(1-e_{2}^{*}\right)\cdots \left(1-e_{n}^{*}\right)} & \frac{e_{2}^{*}\cdots e_{n-1}^{*}}{\left(1-e_{3}^{*}\right)\cdots \left(1-e_{n}^{*}\right)} & \cdots & \frac{e_{n-1}^{*}}{1-e_{n}^{*}} & 1 \end{array}\right]}^{T}.$$ (9)

See Section A.3 for more on the implications of Proposition 2.

**Example 3** Consider again the RFMR in Example 2. Recall that here s* = 1.4268 and e* = [0.1862 0.3539 0.8867]^*T*^. Substituting this in Eq (9) yields *v* = [0.9002 3.1236 1]^*T*^, so (e*)′ = [0.1792 0.6218 0.1991]^T^. This means that if we change the density from s* to $\bar{s}≔s^{*}+\varepsilon$, where *ε* is very small, then the steady-state translation rate changes from R* to

$$\begin{array}{ccc} \bar{R} & = & \lambda_{1}{\bar{e}}_{1}\left(1-{\bar{e}}_{2}\right)\\ & = & \lambda_{1}\left(e_{1}^{*}+\varepsilon {\left(e_{1}^{*}\right)}^{\prime }\right)\left(1-e_{2}^{*}-\varepsilon {\left(e_{2}^{*}\right)}^{\prime }\right)+O\left(\varepsilon^{2}\right)\\ & = & R^{*}+\lambda_{1}\varepsilon \left(\left(1-e_{2}^{*}\right){\left(e_{1}^{*}\right)}^{\prime }-e_{1}^{*}{\left(e_{2}^{*}\right)}^{\prime }\right)+O\left(\varepsilon^{2}\right), \end{array}$$

and substituting the numerical values yields

$$\bar{R}=R^{*}+O\left(\varepsilon^{2}\right).$$

Indeed, this agrees with the fact that the graph of *R*(*s*) attains a maximum at s*.

In Example 2 above the optimal value s* is close, but not equal to one half of the maximal possible total density *n*/2 = 3/2. The next result describes a specific case where the optimal total density is exactly one half of the maximal possible density.

**Proposition 3** *Suppose that the transition rates in the RFMR are symmetric, that is*,

$$\lambda_{i}=\lambda_{n-i},\;i=1,\ldots ,n.$$ (10)

Then s* = *n*/2 *and* $e_{i}^{*}=e_{n+1-i}^{*}$ *for all i*.

Thus, in this case the optimal average density is ρ* = (*n*/2)/*n* = 1/2, and the steady-state occupancies are also symmetric. In other words, when the elongation rates in the RFMR are symmetric the optimal average ribosome density is exactly one half of the maximal possible average ribosome density.

Note that condition (10) always holds for *n* = 2. Also, since a cyclic permutation of the rates leads to an RFMR with the same behavior, it is enough that Eq (10) holds for some cyclic permutation of the rates. For *n* = 3 this holds if at least two of the rates *λ*_1_, *λ*_2_, *λ*_3_ are equal.

We note that a result similar to Proposition 3 is known for the *homogeneous* TASEP with periodic boundary conditions, i.e. that a loading of 50% maximizes the steady-state flow (see, for example, the fundamental diagram in Figure 4.1 in [28]).

### 2.2 Optimal Density in the RFM

Due to the open boundary conditions in the RFM, the number of particles along the chain as a function of time is not conserved. Thus, in this section we analyze the steady-state densities corresponding to the rates that yield a maximal steady-state translation rate. We refer to this density [rates] as the optimal density [rates].

Consider first the problem of maximizing the steady-state translation rate given a constraint on the weighted sum of the initiation and elongation rates. This is motivated by the fact that the biological resources are of course limited. For example, all tRNA molecules are transcribed by the same transcription factors (TFIIIB) and by RNA polymerase III. Hence, if the production of a specific tRNA is increased then the production of some other tRNA must decrease. This is captured by the constraint on the weighted sum of the rates, since any increase in one of the rates must be compensated by a decrease in some other rate. This was formulated in [51] by the following optimization problem.

**Problem 1** *Fix parameters b*, *w*_0_, *w*_1_, …, *w*_*n*_ > 0. *Maximize R* = *R*(*λ*_0_, …, *λ*_*n*_), *with respect to its parameters λ*_0_, …, *λ*_*n*_, *subject to the constraints:*

$$\begin{array}{c} \sum_{i=0}^{n}w_{i}\lambda_{i}\leq b,\\ \lambda_{0},\ldots ,\lambda_{n}\geq 0. \end{array}$$ (11)

In other words, maximize the steady-state protein translation rate given an affine constraint on the initiation and elongation rates. Here *b* is the “total available biocellular budget” that can be spent on all the rates, and the positive values *w*_*i*_, *i* = 0, …, *n*, can be used to provide a different weighting to the different rates.

Problem 1 formalizes, using the RFM, an important problem in both synthetic biology and biotechnology, namely, determine the transition rates that maximize the protein translation rate, given the limited biomolecular budget. See Section A.4 for more details on Problem 1.

Here, our goal is to determine what is the steady-state density when the optimal rates are used (i.e. when we use the rates that maximize the steady-state translation rate under the constraints defined in Problem 1). We refer to this as the optimal density. Let $e_{i}^{*}$, *i* = 1, …, *n*, denote the steady-state density at site *i* corresponding to the optimal rates $\lambda_{0}^{*},\ldots ,\lambda_{n}^{*}$ in Problem 1.

The next example demonstrates that the optimal solution of Problem 1 typically corresponds to a ribosome density that is one half of the maximal possible ribosome density.

**Fig 5:**
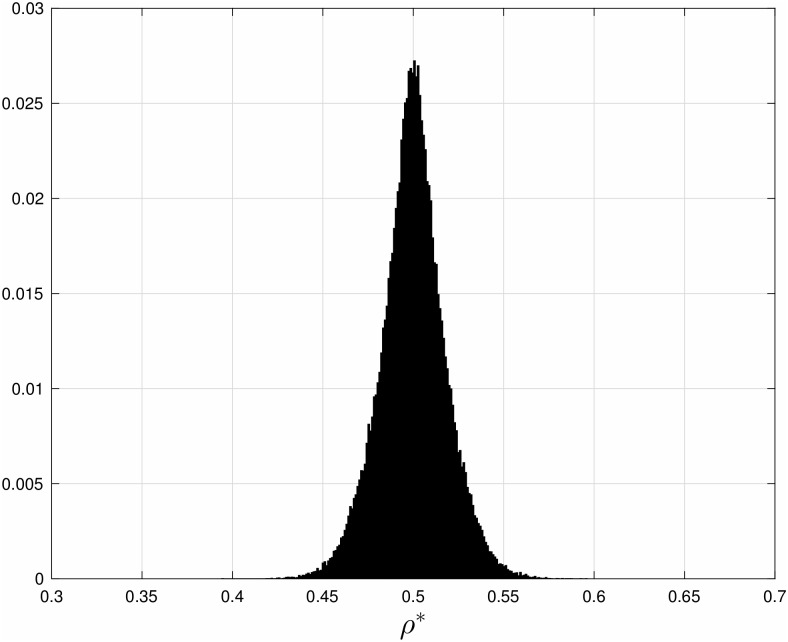
Normalized histogram of the optimal steady-state average density of ribosomes ρ* in Example 4. Data based on 10^5^ random instances of solving the translation maximization problem (Problem 1) for an RFM with length *n* = 11 sites, and total budget *b* = 1 (see Problem 1). It may be observed that typically ρ* is close to 1/2.

**Example 4** Using a simple numerical algorithm we solved 10^5^ instances of Problem 1 for an RFM with length *n* = 11 and total budget *b* = 1. In each instance the weights *w*_*i*_ were drawn independently from a uniform distribution over the interval [0,1]. For each instance, we computed the optimal rates $\lambda_{i}^{*}$ and the corresponding average steady-state optimal density $\rho^{*}≔\frac{1}{n}\sum_{i=1}^{n}e_{i}^{*}$. Fig 5 depicts a normalized histogram (that is, the empirical probability) of the 10^5^ values of ρ*. It may be observed that typically ρ* is close to 1/2. Similar results are obtained when the weights are drawn using other statistics, e.g. exponential, Rayleigh, and Gamma distributions.

In the case where all the weights in Problem 1 are equal we can also derive theoretical results on the structure of the optimal densities and the optimal average density.

#### 2.2.1 Homogeneous Affine Constraint

Recall that in Problem 1 the total weighted sum of the rates is bounded. The different weights can be used to provide a different “importance” for each rate. For example, if *w*_0_ is much larger than the other weights then this means that any small increase in the initiation rate *λ*_0_ will greatly increase the total weighted sum, thus typically forcing the optimal value $\lambda_{0}^{*}$ to be small. In this section we consider the case where all the weights are equal. This means that in the optimization problem all the rates have equal importance. We refer to this case as the *homogeneous constraint* case. Indeed, in this case the weights give equal preference to all the rates, so if the corresponding optimal solution satisfies $\lambda_{i}^{*}>\lambda_{j}^{*}$ for some *i*, *j* then this implies that, in the context of maximizing *R*, *λ*_*i*_ is “more important” than *λ*_*j*_. By Eq (20) (see Appendix A), we may assume in this case, without loss of generality, that *w*_0_ = … = *w*_*n*_ = *b* = 1, so the constraint in Problem 1 becomes

$$\sum_{i=0}^{n}\lambda_{i}\leq 1.$$ (12)

The next result shows that under the homogeneous constraint (12) the steady-state densities corresponding to the optimal solution decrease along the chain, that is the steady-state density at site 1 is the largest, the density at site 2 is the second-largest, etc. It also implies that these steady-state densities are anti-symmetric with respect to the center of the chain. This property immediately implies that the optimal average density is one half (i.e. ρ* = 1/2). In other words, in this case the optimal solution to Problem 1 corresponds to a ribosome density that is one half of the maximal possible density.

**Proposition 4** *Consider Problem 1 with the homogeneous* constraint (12). *Then the optimal steady-state occupancies satisfy*

$$e_{i}^{*}=1-e_{n-i+1}^{*},\;i=1,\ldots ,n.$$ (13)

*If n is even then*

$$e_{1}^{*}>\cdots >e_{\frac{n}{2}}^{*}>\frac{1}{2}>e_{\frac{n}{2}+1}^{*}>\cdots >e_{n}^{*},$$ (14)

*and if n is odd then*

$$e_{1}^{*}>\cdots >e_{\frac{n-1}{2}}^{*}>e_{\frac{n+1}{2}}^{*}=\frac{1}{2}>e_{\frac{n+2}{2}}^{*}>\cdots >e_{n}^{*}.$$ (15)

In both cases, the corresponding optimal density is ρ* = 1/2.

The next example demonstrates the results in Proposition 4.

**Fig 6:**
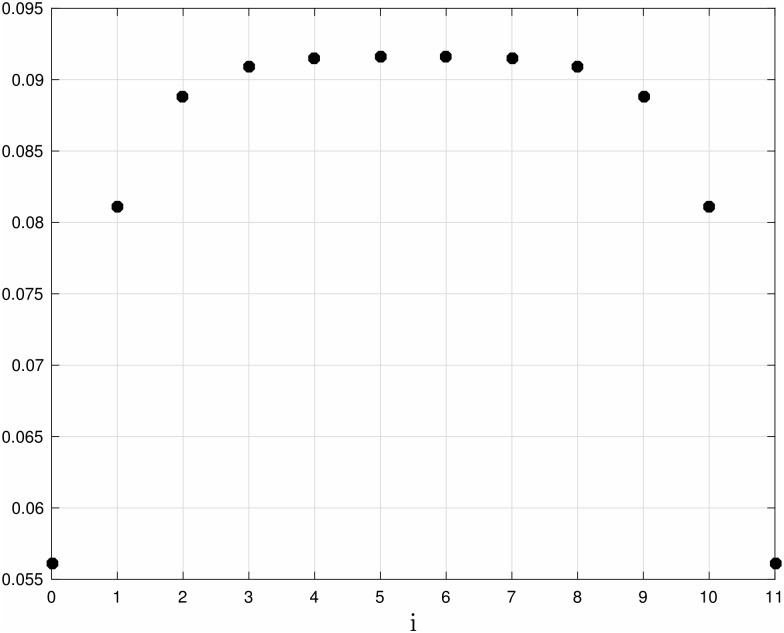
Optimal transition rates $\lambda_{i}^{*}$ as a function of site index *i* for an RFM with *n* = 11 sites. Data obtained by solving the translation maximization problem (Problem 1) with the homogeneous constraint (12). It may be seen that the optimal transition rates $\lambda_{i}^{*}\text{s}$ are symmetric around the center of the chain, with higher values at the center of the chain.

**Fig 7:**
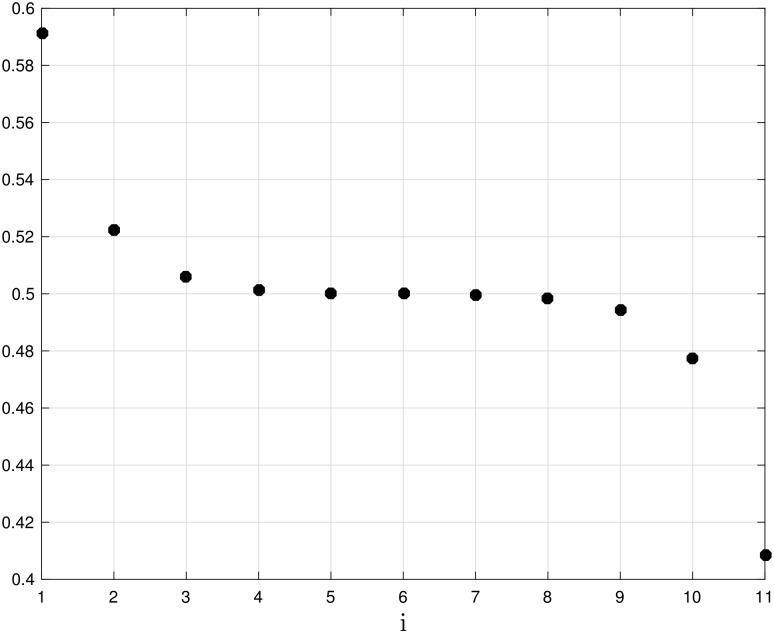
Optimal steady-state ribosome densities $e_{i}^{*}$ as a function of site index *i* for an RFM with *n* = 11 sites. Data obtained by solving the translation maximization problem (Problem 1) with the homogeneous constraint (12). It may be seen that the steady-state densities strictly decrease along the chain and are anti-symmetric with respect to the center of the chain.

**Example 5** Consider Problem 1 for an RFM with *n* = 11 and the homogeneous constraint (12). Fig 6 depicts the optimal values $\lambda_{i}^{*}$, *i* = 0, …, 11. It may be seen that the $\lambda_{i}^{*}\text{s}$ are symmetric, i.e. $\lambda_{i}^{*}=\lambda_{11-i}^{*}$, and that they increase towards the center of the chain. The corresponding steady-state distribution is e* = [0.5913, 0.5224, 0.5059, 0.5016, 0.5004, 0.5000, 0.4996, 0.4984, 0.4941, 0.4776, 0.4087]^*T*^ (see Fig 7). It may be seen that the steady-state densities strictly decrease along the chain and are anti-symmetric with respect to the center of the chain.

Since the RFM is the dynamic mean-field approximation of TASEP, our results naturally raise the question of what is the optimal particle density in TASEP, that is, the density yielding the maximal possible output rate. Recall that each particle models a ribosome and each ribosome that leaves the chain produces a protein, so the output rate is also the protein translation rate. Rigorous analysis of this problem in TASEP seems to be non trivial.

We used a simple grid-search to address the problem of maximizing the steady-state flow in HTASEP (i.e. TASEP with all internal rates equal to one) with respect to the parameters *α* and *β* subject to the constraint *w*_1_*α* + *w*_2_ *β* = *b*. For *L* = 11 and *w*_1_ = *w*_2_ = *b* = 1 the solution is α* = β* = 1/2, and the corresponding steady-state occupancies (computed using Eq (3.65) in [30]) are all equal to 1/2. Thus the average optimal density is ρ* = 1/2.

**Fig 8:**
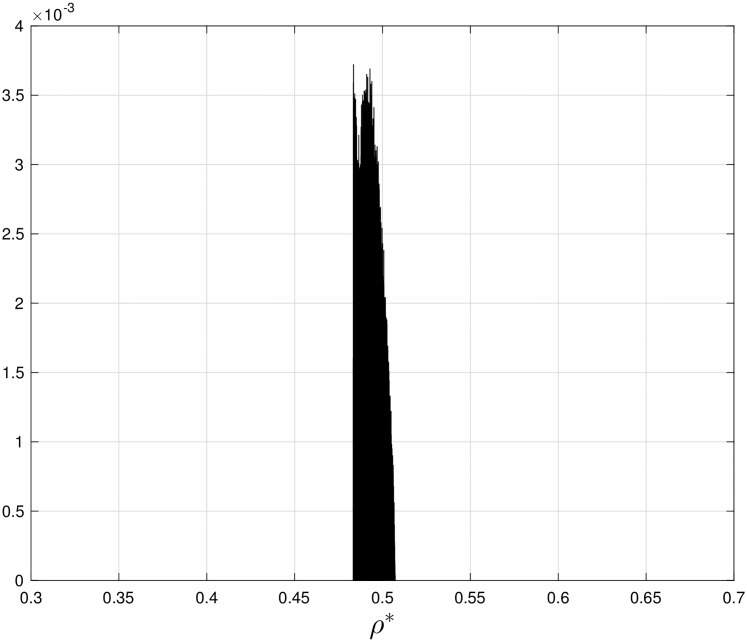
Normalized histogram of the average steady-state optimal ribosome density ρ* in HTASEP with *L* = 30 sites, and stochastic rates that maximize the translation rate. It may be seen that ρ* is typically close to 1/2, collaborating the analytic results derived for the RFM.

We also ran 10000 tests with *w*_1_ and *w*_2_ chosen from an independent uniform distribution on [0,1]. In each case, a simple grid-search was used to find the optimal rates. Fig 8 depicts a normalized histogram of the optimal average steady-state ribosome density in an HTASEP with *L* = 30. It may be seen that the typical optimal density is about 1/2. A similar result has been reported in [58] that used HTASEP with a superposition of open and periodic boundary conditions.

These simulation results corroborate the analytic results derived above for the RFM and RFMR.

## 3 Discussion

A natural analogy for the cell is that of a factory operating complex and inter-dependent biosynthesis assembly processes [59]. Increasing the translation rate can be done by both operating several identical processes in parallel, and by pipelining every single process. In the context of translation, many mRNA copies of the same gene are translated in parallel, and the same transcript is simultaneously translated by several ribosomes. A natural question is what is the density of ribosomes along the transcript that leads to a *maximal* translation rate. It is clear that a very small density will not be optimal, and since the ribosomes interact and may jam each other, a very high density is also not optimal.

We studied this question using dynamical models for ribosome flow in both a linear and a circular mRNA molecule. Our results show that typically the optimal density is close to one half of the maximal possible density.

In synthetic biology and biotechnology optimizing the translation rate is a standard goal, and we believe that our results can provide guidelines for designing and re-engineering transcripts. However, in vivo biological regulation of mRNA translation may have several goals besides optimizing the translation rate. For endogenous genes there are many additional constraints that shape the transcript, translation rates, and ribosome densities. For example, it is known that evolution optimizes not only protein levels, but also attempts to minimize their production cost [60, 61]. This cost may include for example the biocellular budget required for producing the ribosomes themselves. Thus, we do not expect that the protein levels of all genes will be maximal. Rather, we expect that translation is optimized for proteins that are required with high copy numbers (e.g. those related to house keeping genes and some structural genes).

Furthermore, it is important to mention that there are various additional constraints shaping the coding regions of endogenous genes. These include various regulatory signals related to various gene expression steps, co-translational folding, and the functionality of the protein [22, 23, 62–65]. Thus, under these additional constraints we do not necessarily expect to see ribosome densities that maximize the translation rate.

Indeed, experimental studies of ribosome densities in various organisms demonstrate that on average 15% − 20% of the mRNA is occupied by ribosomes [66, 67]. However, in 241 genes in *S. cerevisiae* more than 40% of the mRNA is occupied by ribosomes [66]. This suggests that a ribosome density that is close to 1/2 is frequent in certain *specific* mRNA molecules. In addition, it seems that under stress conditions ribosomal densities (and traffic jams) increase (see, e.g. [17]). Thus, under such conditions we expect more mRNAs with ribosome densities close to 1/2 (see, for example, [68]).

Interestingly, the reported results are also in agreement with genome-wide simulations of the RFM that were performed based on the modeling of all the endogenous genes of *S. cerevisiae*, as reported in [29]. Indeed, Fig 4C there shows the ribosome density, averaged over all the sites of all the mRNAs, as a function of the initiation rate. The maximal translation rate corresponds to an average density of about 1/2.

We note in passing that for an RFM with dimension *n*, with all the rates equal (i.e. *λ*_0_ = … = *λ*_*n*_), the average steady-state ribosomal density is 1/2 for all *n*, and that for an RFM with dimension *n*, *λ*_0_ → ∞, and equal elongation rates (i.e. *λ*_1_ = … = *λ*_*n*_), the average steady-state ribosomal density is $\frac{n+1}{2n}$, thus approaching 1/2 as *n* increases [69].

Further studies may consider optimizing the translation rate under various additional constraints. For example, it will be interesting to study the optimal ribosome density when taking into account also the biocellular cost of protein production, or under given constraints on the allowed density profile, etc. In addition, it will be interesting to study the optimal densities in more comprehensive models that include competition for the free ribosomes between several mRNA molecules [57]. Another important issue, that is not captured by the RFM and RFMR, is that every ribosome covers several codons. Developing and analyzing RFM/RFMR models with “extended objects” is an important challenge.

Finally, TASEP has been used to model and analyze many other natural and artificial processes including traffic flow and the movement of motor proteins. The problem of the optimal density is of importance in these applications as well.

## A Appendix: Mathematical Background and Details

### A.1 RFM

Let *x*(*t*, *a*) denote the solution of Eq (1) at time *t* ≥ 0 for the initial condition *x*(0) = *a*. Since every state variable *x*_*i*_ describes the occupation level at site *i* normalized between zero and one, this may also be interpreted as the probability that site *i* contains a ribosome. We always assume that *a* belongs to the closed *n*-dimensional unit cube: $C^{n}≔\{x\in {\mathbb{R}}^{n}:x_{i}\in [0,1],i=1,\ldots ,n\}.$ It is straightforward to verify that this implies that *x*(*t*, *a*) ∈ *C*^*n*^ for all *t* ≥ 0. In other words, *C*^*n*^ is an invariant set of the dynamics [48]. This means that the occupancy levels always remain bounded between zero and one.

At steady-state, that is for *x* = *e* the left-hand side of all the equations in Eq (1) is zero, so

$$\begin{array}{ccc} \lambda_{0}\left(1-e_{1}\right) & = & \lambda_{1}e_{1}\left(1-e_{2}\right)\\ & = & \lambda_{2}e_{2}\left(1-e_{3}\right)\\ & \vdots & \\ & = & \lambda_{n-1}e_{n-1}\left(1-e_{n}\right)\\ & = & \lambda_{n}e_{n}\\ & = & R. \end{array}$$ (16)

This yields the following expressions for the final ribosome density profile:

$$\begin{array}{ccc} e_{n} & = & R/\lambda_{n},\\ e_{n-1} & = & R/\left(\lambda_{n-1}\left(1-e_{n}\right)\right),\\ & \vdots & \\ e_{2} & = & R/\left(\lambda_{2}\left(1-e_{3}\right)\right),\\ e_{1} & = & R/\left(\lambda_{1}\left(1-e_{2}\right)\right), \end{array}$$ (17)

and

$$e_{1}=1-R/\lambda_{0}.$$ (18)

Combining Eqs (17) and (18) provides an elegant *finite continued fraction* [70] expression for the steady-state translation rate *R* in terms of the initiation and elongation rates along the coding region:

0=1-R/λ01-R/λ11-R/λ2aaaaaaa⋱1-R/λn-11-R/λn. (19)

Note that this equation admits several solutions for *R*, however, we are interested only in the unique feasible solution, i.e. the solution corresponding to *e* ∈ Int(*C*^*n*^). Indeed, this is the only solution that also yields a value between zero and one for the steady-state ribosome density at each site.

Note also that Eq (19) implies that

$$R\left(c\lambda_{0},\ldots ,c\lambda_{n}\right)=cR\left(\lambda_{0},\ldots ,\lambda_{n}\right),\;\text{for}\;\text{all}\;c>0,$$ (20)

that is, *R*(*λ*_0_, …, *λ*_*n*_) is a *homogeneous function* of degree one. This means that if the initiation rate and all the translation elongation rates are multiplied by a factor *c* > 0 then the steady-state translation rate will also increase by a factor *c*.

Ref. [51] proved that *R*(*λ*_0_, …, *λ*_*n*_) is a *strictly concave* function on $R_{++}^{n+1}$. Please see the figures in [51] for an intuitive explanation of this property.

### A.2 RFMR

Eq (4) implies that

$$\frac{d}{dt}H\left(x\left(t\right)\right)\equiv 0,\;\text{for}\;\text{all}\;t\geq 0,$$

so the total ribosome density is conserved, i.e.

H(x(t))=H(x(0)),forallt≥0. (21)

Let *R* = *R*(*s*, *λ*_1_, …, *λ*_*n*_) denote the steady-state translation rate in the RFMR for any *x*(0)∈*L*_*s*_. It follows from Eq (4) that *R* = *λ*_*i*_ *e*_*i*_(1 − *e*_*i* + 1_), *i* = 1, …, *n* (recall that in the RFMR, every index is interpreted modulo *n*). It is straightforward to verify that for any *c* > 0

$$R\left(s,c\lambda_{1},\ldots ,c\lambda_{n}\right)=cR\left(s,\lambda_{1},\ldots ,\lambda_{n}\right).$$ (22)

This means that if the initiation rate and all the translation elongation rates are multiplied by a factor *c* > 0 then the protein translation rate will also increase by a factor *c*.

### A.3 Optimal Density in the RFMR

In order to better understand Fig 4 of Example 2, note that the equilibrium point in the RFMR satisfies:

$$e_{1}+\ldots +e_{n}=s,$$

and, by Eq (4),

$$\begin{array}{ccc} \lambda_{n}e_{n}\left(1-e_{1}\right) & = & \lambda_{1}e_{1}\left(1-e_{2}\right),\\ & = & \lambda_{2}e_{2}\left(1-e_{3}\right),\\ & \vdots & \\ & = & \lambda_{n-1}e_{n-1}\left(1-e_{n}\right). \end{array}$$ (23)

For any *i* ∈ {1, …, *n*}, let *k*_*i*_ ≔ *λ*_1_…*λ*_*i* − 1_ *λ*_*i* + 1_…*λ*_*n*_, i.e. the product of all the elongation rates except for rate *i*, and let $\mu ≔\sum_{i=1}^{n}\lambda_{i}$, that is, the sum of all the elongation rates. If *s* ≈ 0 then all the *e*_*i*_s will be small, so we can ignore the terms 1 − *e*_*i*_ in Eq (23), and this yields $e_{i}\approx \frac{k_{i}s}{\mu }$, *i* = 1, …, *n*. A similar argument shows that if *s* ≈ *n* then $e_{i}\approx 1-\frac{k_{i-1}\left(n-s\right)}{\mu }$, *i* = 1, …, *n*. For the particular case in Example 2 this implies that when *s* ≈ 0 then *e* ≈ (*s*/22) [3 1 18]^*T*^. In particular, *e*_2_ < *e*_1_ < *e*_3_. When *s* ≈ 3 then *e* ≈ (*s*/22) [18*s* − 32 3*s* + 13 *s* + 19]^*T*^. In particular, *e*_1_ < *e*_2_ < *e*_3_. In other words, the relative ordering between the steady-state density at each site may change as the total density changes.

Regrading Proposition 2, it also implies the following. Indeed it follows from Eqs (9) and (5) that $\frac{v_{i}}{v_{i-1}}=\frac{1-e_{i}^{*}}{e_{i-1}^{*}}$, *i* = 1, 2, …, *n*. This means that *v*_*i*_ > *v*_*i* − 1_ if and only if $1-e_{i}^{*}>e_{i-1}^{*}$. In other words, the sensitivity to a change in the total optimal density at site *i* is larger than the sensitivity at site *i* − 1 if and only if the amount of “free space” at site *i* is larger than the occupancy at site *i* − 1.

### A.4 Optimal Density in the RFM

Consider Problem 1. It has been shown in [51] that the optimal solution $\lambda_{0}^{*},\ldots ,\lambda_{n}^{*}$ always satisfies $\sum_{i=0}^{n}w_{i}\lambda_{i}^{*}=b$. Of course, by scaling the *w*_*i*_s we may always assume that *b* = 1. Combining this with the strict concavity of the steady-state translation rate *R*(*λ*_0_, …, *λ*_*n*_) in the RFM implies that Problem 1 is a *convex optimization problem* that admits a unique optimal solution $\lambda^{*}\in R_{++}^{n+1}$. Also, this solution can be determined efficiently using numerical algorithms that scale well with *n*.

## B Appendix: Proofs

*Proof of Proposition 1*. It follows from known results on the solutions of ODEs that *e*_*i*_ is continuous in *s* for all *i*. It is known that every *e*_*i*_ is strictly increasing in *s* (see [Theorem 1] in [31]). Hence, there exists a set *E* of measure zero such that for all *i* and all *s* ∈ [0,*n*] \ *E* the derivative $e_{i}^{\prime }≔\frac{d}{ds}e_{i}$ exists, and is strictly positive. The steady-state translation rate satisfies *R* = *λ*_*i*_ *e*_*i*_(1 − *e*_*i* + 1_), for all *i* = 1, …, *n*. This yields

$$R^{\prime }=\lambda_{i}\left(e_{i}^{\prime }\left(1-e_{i+1}\right)-e_{i}e_{i+1}^{\prime }\right),$$ (24)

for all *i* and all *s* ∈ [0,*n*] \ *E*.

Let sgn(·):R→{-1,0,1} denote the sign function, i.e.

$$\text{sgn}\left(y\right)=\left\{\begin{array}{cc} 1, & y>0,\\ 0, & y=0,\\ -1, & y<0. \end{array}\right.$$

We require the following result.

**Proposition 5** *For any s* ∈ [0,*n*] \ *E*,

$$\text{sgn}\left(R^{\prime }\right)=\text{sgn}\left(\prod_{i=1}^{n}\left(1-e_{i}\right)-\prod_{i=1}^{n}e_{i}\right).$$

*Proof of Proposition 5*. Assume that *R*′ > 0. Then Eq (24) yields

$$e_{i}^{\prime }\left(1-e_{i+1}\right)>e_{i}e_{i+1}^{\prime },\;i=1,\ldots ,n.$$

Multiplying these *n* inequalities, and using the fact that $e_{i}^{\prime }>0$ for all *i* yields

$$\prod_{i=1}^{n}\left(1-e_{i}\right)>\prod_{i=1}^{n}e_{i}.$$ (25)

To prove the converse implication, assume that Eq (25) holds. Multiplying both sides of the inequality by the strictly positive term $\prod_{j=1}^{n}e_{i}^{\prime }$ yields

$$\prod_{i=1}^{n}a_{i}>\prod_{i=1}^{n}b_{i},$$

where $a_{i}≔e_{i}^{\prime }\left(1-e_{i+1}\right)$, and $b_{i}≔e_{i}e_{i+1}^{\prime }$. This means that *a*_ℓ_ > *b*_ℓ_ for some index ℓ ∈ {1, …, *n*}. Since *R*′ = *λ*_ℓ_(*a*_ℓ_ − *b*_ℓ_), it follows that *R*′ > 0. Thus, we showed that *R*′ > 0 if and only if $\prod_{i=1}^{n}\left(1-e_{i}\right)>\prod_{i=1}^{n}e_{i}$. The proof that *R*′ < 0 if and only if $\prod_{i=1}^{n}\left(1-e_{i}\right)<\prod_{i=1}^{n}e_{i}$ is similar. This implies that *R*′ = 0 if and only if $\prod_{i=1}^{n}\left(1-e_{i}\right)=\prod_{i=1}^{n}e_{i}$, and this completes the proof of Proposition 5.

We can now complete the proof of Proposition 1. Let $p\left(s\right)≔\prod_{i=1}^{n}\left(1-e_{i}\right)$, and $q\left(s\right)≔\prod_{i=1}^{n}e_{i}$. Then *p*(0) = 1, *p*(*n*) = 0, *q*(0) = 0, and *q*(*n*) = 1. The strict monotonicity of every *e*_*i*_ implies that *p*(*s*) [*q*(*s*)] is a strictly decreasing [increasing] function in the interval *s* ∈ [0,*n*]. This implies that there is a unique s* ∈ [0,*n*] such that *p*(s*) = *q*(s*). By Proposition 5, this is the unique maximizer of *R*(*s*), and for *s* = s*:

$$e_{1}^{*}\ldots e_{n}^{*}=\left(1-e_{1}^{*}\right)\ldots \left(1-e_{n}^{*}\right).$$ (26)

Also,

$$\begin{array}{ccc} R^{*} & = & \lambda_{1}e_{1}^{*}\left(1-e_{2}^{*}\right)\\ & = & \lambda_{2}e_{2}^{*}\left(1-e_{3}^{*}\right)\\ & \vdots & \\ & = & \lambda_{n}e_{n}^{*}\left(1-e_{1}^{*}\right), \end{array}$$ (27)

and this yields ${\left(R^{*}\right)}^{n}=\left(\lambda_{1}\ldots \lambda_{n}\right)\left(e_{1}^{*}\ldots e_{n}^{*}\right)\left(\left(1-e_{1}^{*}\right)\ldots \left(1-e_{n}^{*}\right)\right)$. Using Eq (26) completes the proof of Proposition 1.

*Proof of Fact 1*. For *n* = 2, Eq (26) yields $e_{1}^{*}+e_{2}^{*}=1$, and substituting this in Eq (27) yields Eq (7). Consider the case *n* = 3. Let *λ* ≔ *λ*_1_ *λ*_2_ *λ*_3_. It follows from Eq (27) that

$$\begin{array}{c} \lambda_{2}\lambda_{3}R^{*}=\lambda e_{1}^{*}\left(1-e_{2}^{*}\right),\\ \lambda_{1}\lambda_{3}R^{*}=\lambda e_{2}^{*}\left(1-e_{3}^{*}\right),\\ \lambda_{1}\lambda_{2}R^{*}=\lambda e_{3}^{*}\left(1-e_{1}^{*}\right). \end{array}$$

Summing these equations yields

$$\eta R^{*}=\lambda s^{*}-\lambda \left(e_{1}^{*}e_{2}^{*}+e_{2}^{*}e_{3}^{*}+e_{3}^{*}e_{1}^{*}\right),$$ (28)

where *η* ≔ *λ*_2_ *λ*_3_ + *λ*_1_ *λ*_3_ + *λ*_1_ *λ*_2_. It follows from Eq (26) that

$$e_{1}^{*}e_{2}^{*}+e_{2}^{*}e_{3}^{*}+e_{3}^{*}e_{1}^{*}=s^{*}-1+2e_{1}^{*}e_{2}^{*}e_{3}^{*},$$

and substituting this in Eq (28) yields $\eta R^{*}=\lambda \left(1-2e_{1}^{*}e_{2}^{*}e_{3}^{*}\right)$. Applying Eq (6) completes the proof.

*Proof of Proposition 2*. Write Eq (24) as

$$D{\left(e^{*}\right)}^{\prime }=C{\left(e^{*}\right)}^{\prime },$$ (29)

where $D≔\text{diag}(1-e_{2}^{*},1-e_{3}^{*},\ldots ,1-e_{n}^{*},1-e_{1}^{*})$, and

$$C≔\left[\begin{array}{ccccccc} 0 & e_{1}^{*} & 0 & 0 & \ldots & 0 & 0\\ 0 & 0 & e_{2}^{*} & 0 & \ldots & 0 & 0\\ & & \vdots \\ 0 & 0 & 0 & 0 & \ldots & 0 & e_{n-1}^{*}\\ e_{n}^{*} & 0 & 0 & 0 & \ldots & 0 & 0 \end{array}\right].$$

Note that *C* is cyclic of order *n*, so multiplying Eq (29) by *C*^*n*−1^ yields

$$H{\left(e^{*}\right)}^{\prime }=\left(e_{1}^{*}\ldots e_{n}^{*}\right){\left(e^{*}\right)}^{\prime },$$ (30)

where *H* ≔ *C*^*n*−1^ *D*. In other words, (e*)′ is an eigenvector of *H* corresponding to the eigenvalue $\left(e_{1}^{*}\ldots e_{n}^{*}\right)$. The cyclic structure of *C* implies that

$$C^{n-1}=\left[\begin{array}{ccccc} 0 & 0 & \ldots & 0 & \mu_{1}^{*}\\ \mu_{2}^{*} & 0 & \ldots & 0 & 0\\ & & \vdots \\ 0 & \ldots & \mu_{n-1}^{*} & 0 & 0\\ 0 & 0 & \ldots & \mu_{n}^{*} & 0 \end{array}\right],$$

where $\mu_{i}^{*}≔e_{i}^{*}e_{i+1}^{*}\ldots e_{i+n-2}^{*}$, with all indexes interpreted modulo *n* (e.g., $e_{n+1}^{*}=e_{1}^{*}$). Now it is straightforward to verify that (e*)′ = *cv*, with *c* ≠ 0, is the only solution of Eq (30). Since every *e*_*i*_ increases with *s*, we conclude that *c* > 0. Furthermore, $\sum_{i=1}^{n}e_{i}^{*}=s$ implies that $\sum_{i=1}^{n}{\left(e_{i}^{*}\right)}^{\prime }=1$, and this completes the proof.

*Proof of Proposition 3.* The proof follows immediately from the following result.

**Proposition 6** *Consider an RFMR with dimension n, and suppose that the transition rates satisfy λ*_*i*_ = *λ*_*n*−*i*_ *for all i. Then*

1. $e_{i}^{*}=e_{n+1-i}^{*}$ *for any i*;
2. *R*(*s*) = *R*(*n* − *s*) for any *s* ∈ [0,*n*], and *R*(*s*_1_) < *R*(*s*_2_) *for any* 0 ≤ *s*_1_ < *s*_2_ ≤ *n*/2.

This means in particular that *R*(*s*) is symmetric with respect to *s* = *n*/2, and is strictly increasing in the interval [0,*n*/2).

*Proof of Proposition 6*. Given an RFMR with dimension *n*, and rates *λ*_*i*_, *i* = 1, …, *n*, let ${\bar{x}}_{i}\left(t\right)≔1-x_{n+1-i}\left(t\right)$, *i* = 1, …, *n*. Then using the equation

$${\dot{x}}_{i}=\lambda_{i-1}x_{i-1}\left(1-x_{i}\right)-\lambda_{i}x_{i}\left(1-x_{i+1}\right)$$

yields

$${\dot{\bar{x}}}_{i}={\bar{\lambda }}_{i-1}{\bar{x}}_{i-1}\left(1-{\bar{x}}_{i}\right)-{\bar{\lambda }}_{i}{\bar{x}}_{i}\left(1-{\bar{x}}_{i+1}\right),$$

with ${\bar{\lambda }}_{i}≔\lambda_{n-i}$ (recall that all indexes are interpreted modulo *n*). This is again an RFMR. Fix an arbitrary *s* ∈ [0,*n*]. Then for any *x*(0) such that $1_{n}^{T}x\left(0\right)=s$ we have $1_{n}^{T}\bar{x}\left(0\right)=n-s$. Therefore, the *x* system converges to *e* = *e*(*s*, *λ*_1_, …, *λ*_*n*_), and the $\bar{x}$ system to $\bar{e}=e\left(n-s,{\bar{\lambda }}_{1},\ldots ,{\bar{\lambda }}_{n}\right)$. This implies that $e_{i}\left(s,\lambda_{1},\ldots ,\lambda_{n}\right)=1-e_{n+1-i}\left(n-s,{\bar{\lambda }}_{1},\ldots ,{\bar{\lambda }}_{n}\right)$ for all *i*. The steady-state translation rate in the $\bar{x}$ system is

$$\begin{array}{ccc} \bar{R} & = & {\bar{\lambda }}_{n}{\bar{e}}_{n}\left(1-{\bar{e}}_{1}\right)\\ & = & \lambda_{n}\left(1-e_{1}\right)e_{n}\\ & = & R. \end{array}$$

If the rates satisfy *λ*_*i*_ = *λ*_*n*−*i*_ for all *i* then *e*_*i*_(*s*) = 1 − *e*_*n*+1 − *i*_(*s*) for all *i*, and *R*(*s*) = *R*(*n* − *s*). By Proposition 1, this means that R* = *R*(*n*/2). Combining this with the results in the proof of Proposition 1 completes the proof of Proposition 6.

*Proof of Proposition 4*. Consider Problem 1 and the homogeneous constraint (12). By [Proposition 4] in [71]:

$$e_{i}^{*}=1-e_{n-i+1}^{*},$$ (31)

and

$$\frac{\lambda_{i}^{*}}{\lambda_{i-1}^{*}}=\frac{e_{i}^{*}}{1-e_{i}^{*}},$$ (32)

*i* = 1, …, *n*, and by [Theorem 1] in [71]:

$$\lambda_{0}^{*}<\lambda_{1}^{*}<\ldots <\lambda_{\left\lfloor n/2\right\rfloor }^{*},$$ (33)

and

$$\lambda_{i}^{*}=\lambda_{n-i}^{*},\;i=0,\ldots ,n.$$ (34)

Thus, Eq (31) proves Eq (13), and combining Eqs (33), (34) and (32) yields Eqs (14) and (15).
