# Correction: On the Ribosomal Density that Maximizes Protein Translation Rate

**DOI:** 10.1371/journal.pone.0177650

**Published:** 2017-05-09

**Authors:** Yoram Zarai, Michael Margaliot, Tamir Tuller

There is an error in the equation under equation (10) of Proposition 3 under the “2.1 Optimal Density in the RFMR” subheading of the “2 Main Results” section. The correct equation is: $s^{*}=\frac{n}{2}\;and\;{e^{*}}_{i}=1-{e^{*}}_{n+1-i}$ for all i.

There is an error in the first equation of Proposition 6 in the “B Appendix: Proofs” section. The correct equation is e^*^_i_ = 1 − e^*^_n+1−*i*_ for any i.
